# Structural and Functional Analysis of Multi-Interface Domains

**DOI:** 10.1371/journal.pone.0050821

**Published:** 2012-12-14

**Authors:** Liang Zhao, Steven C. H. Hoi, Limsoon Wong, Tobias Hamp, Jinyan Li

**Affiliations:** 1 School of Computer Engineering, Nanyang Technological University, Singapore, Singapore; 2 School of Computing, National University of Singapore, Singapore, Singapore; 3 Technische Universitaet Muenchen, Bioinformatics-I12, Informatik, Muenchen, Germany; 4 Advanced Analytics Institute, University of Technology Sydney, Sydney, Australia; MRC National Institute for Medical Research, United Kingdom

## Abstract

A multi-interface domain is a domain that can shape multiple and distinctive binding sites to contact with many other domains, forming a hub in domain-domain interaction networks. The functions played by the multiple interfaces are usually different, but there is no strict bijection between the functions and interfaces as some subsets of the interfaces play the same function. This work applies graph theory and algorithms to discover fingerprints for the multiple interfaces of a domain and to establish associations between the interfaces and functions, based on a huge set of multi-interface proteins from PDB. We found that about 40% of proteins have the multi-interface property, however the involved multi-interface domains account for only a tiny fraction (1.8%) of the total number of domains. The interfaces of these domains are distinguishable in terms of their fingerprints, indicating the functional specificity of the multiple interfaces in a domain. Furthermore, we observed that both cooperative and distinctive structural patterns, which will be useful for protein engineering, exist in the multiple interfaces of a domain.

## Introduction

**Figure 1:**
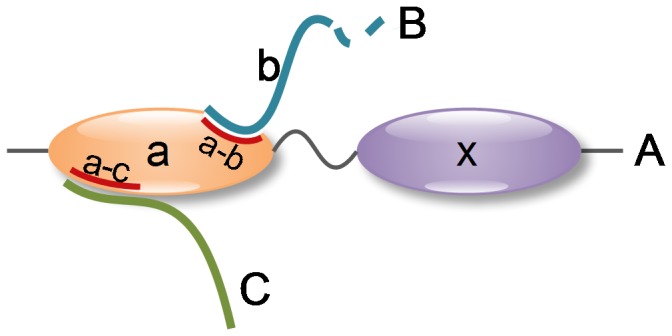
Multi-interface domain illustration. Domain *a* of protein *A* interacting with domain *b* of protein *B* produces interface *a–b* on domain *a*, and domain *a* binding to protein *C* generates interface *a–c* on domain *a*. Interfaces *a–b* and *a–c* are distinguishable on domain *a*, thus domain *a* is a multi-interface domain.

**Figure 2:**
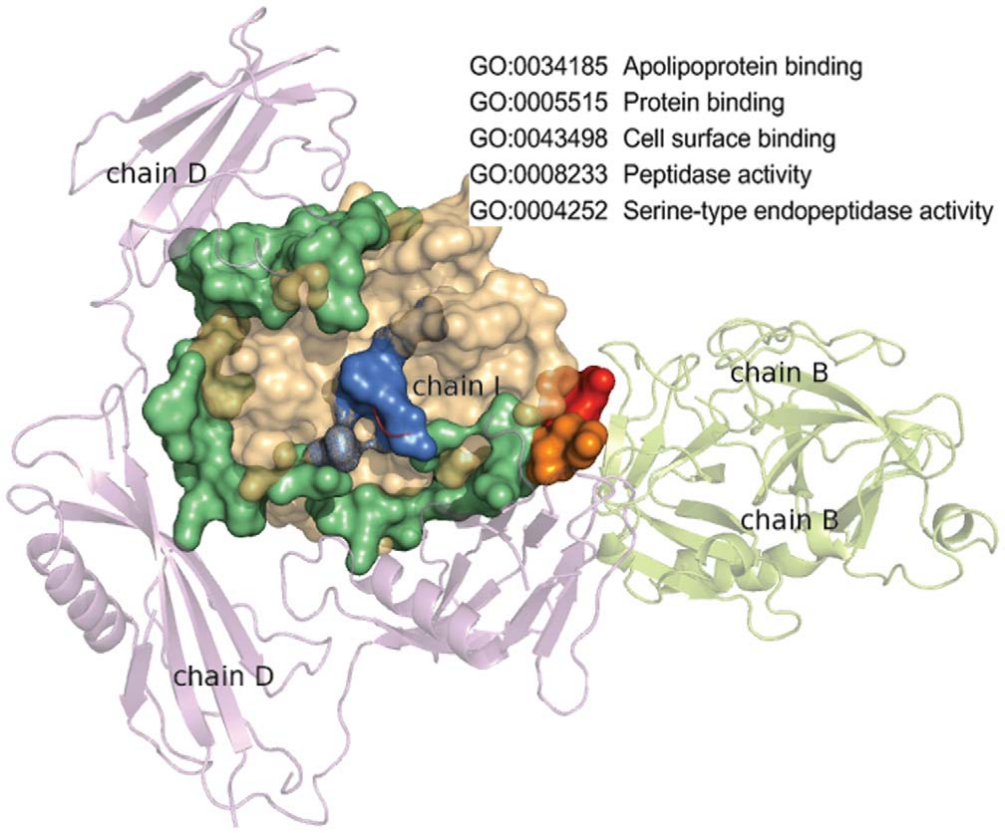
Multiple interfaces in the catalytic domain of plasmin. The interfaces are colored in limegreen (46 residues), marine (6 residues), and red (7 residues) for plasmin interacting with a streptokinase, a protein inhibitor, and another plasmin symmetric unit, respectively. The two overlapping residues are colored orange, and the five molecular functions of this domain retrieved from GO are shown at the top right corner.

A protein domain is usually a contiguous segment in a protein's primary sequence that can be independently folded to form a stable tertiary structure. Domains vary in length from about 25 amino acids to about 500 amino acids. The number of domains is huge—for example, there are 110,800 domains currently stored in the Structural Classification of Proteins (SCOP) database [1]. As building block of proteins, each domain can be used by a variety of different proteins. When two proteins A and B have an interaction, it is usually an interaction between some domain 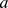 of A and some domain 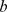 of B. The domain 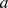 of protein A may have an interaction with protein C at a different binding site as shown in Figure 1. Thus, there can be multiple interfaces in a domain. Those domains with multiple interfaces are defined as “multi-interface domains”. Related concepts of multi-interface have been proposed elsewhere previously. For example, “multi-interface hub”, as defined by Kim, is used to illustrate a protein interacting with multiple proteins synchronously or asynchronously [2], “multibinding protein interface”, as defined by Tyagi, is to describe the same interface with interactions to several partners asynchronously [3], and “multi-ligand interface”, as defined by Dasgupta, is to depict the union of overlapping interfaces on a protein [4]. Figure 2 shows an example of an multi-interface domain where three different interfaces of the catalytic domain of plasmin are presented. This domain in fact has thirteen different interfaces playing five molecular functions according to Gene Ontology (GO) annotations [5].

Multi-interface domains can be found in the following situations: (i) One domain of a protein interacts with multiple other proteins at the same time. For example, as mentioned above, the catalytic domain of plasminogen can interact with three other plasmins simultaneously to shape three non-overlapping interfaces. (ii) The same domain of a protein interacts with other different proteins at different time or environments. For instance, an antigen peptide can interact with a major histocompatibility complex (MHC) molecule, while it can be also recognized by a T lymphocyte after being presented to the cell surface by an MHC molecule. This phenomenon is previously highlighted by Narayan *et al.* [6] as well, in a discussion on the multiple binding sites of the interferon-regulated transcription factor IRF-1, which is involved in several biological processes such as antiviral response and tumor suppression. (iii) Multiple copies 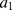, 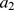, …, 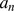 of a domain 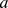 are included in different proteins 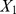, 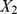, …, 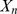, and each of the 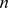 copies of the domain 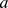 has an interaction with another protein at a different binding site. For example, domain D-maltodextrin-binding protein is contained in several proteins, such as in maltose-binding periplasmic (MBP) (PDB ID 3LC8), in MBP/NEDD8-activating enzyme compound 10 E1 catalytic subunit chimera (PDB ID 2NVU) and in maltose binding-A1 homeodomain protein chimera (PDB ID 1MH4). The aforementioned three situations have unique feature on their own although they are similar to each other. The first two situations are classified based on temporal information, while the third one—distinct from the first two—is based on genetic information.

The multiple interfaces in a domain can be grouped into subsets such that each of them share a unique biological function. These functions are usually distinguishable and non exchangeable. As an example shown in Figure 2, the three functions of the plasmin catalytic domain cannot be swapped with regard to their interfaces. More interestingly, sometimes the number of such subsets of the interfaces in a domain can be large. As protein functions are currently annotated at the domain level, such as those by GO [5], it is difficult to figure out which interface in a domain possesses what function based on the current annotations. The study of multi-interface domains can associate interfaces with their functions more precisely. Yet almost all past studies of proteins are either at the protein level (*e.g.*, protein-protein interaction, protein complex identification) [7], [8], or at the domain level (*e.g.*, domain-domain interaction, domain transitivity analysis) [9], [10], or at residue level (*e.g.*, interface residue identification, hot spots prediction) [11], [12]. Studies are seldom undertaken at the interface level, which is in the middle between the domain and residue levels. This is probably attributable to uncertainties in locating an interface due to the adaptation, context-awareness, or re-configuration of interfaces [6], [13], [14], in contrast to the clear boundaries possessed by a protein, domain or residue. Some notable exceptions are the works of identifying conserved interface patterns by interface alignment [15], detecting common 3D sites in proteins by frequent graph patterns of stereochemical atom groups [16], uncovering functional sites in protein families by recurring graph patterns [17], and delineating biological functions of proteins by common atomic motifs of interfaces [18], [19]. However, all these methods mix up interfaces from different domains even if they are remarkably different. In addition, multiple interfaces in one domain are deemed as independent as no relation has been unveiled. Further more, associations between multiple interfaces and their biological functions still remain unanswered.

In this study, we address the following questions: (i) What kind of domains prefer the multi-interface property? That is, we want to know the distribution of domains that have multiple interfaces. (ii) What are the fingerprints of an interface, or a subset of interfaces, in a domain? That is, we want to discover unique structures in a domain that distinguish the multiple interfaces from each other. (iii) What are the relationships between the multiple interfaces in a domain? That is, we want to see whether the multiple interfaces in a domain have any cooperative or competitive relations. (iv) What are the associations between multiple interfaces and their molecular functions?

Our study thus aims to unveil some facets of protein interaction mechanisms by looking at the multiple interfaces of a domain. This is new because past interface analysis [20]–[22] generally provides only generic profiles of interface residue organization (e.g., residues contacting graph, hydrophobicity distribution, polarity scattering) and interface residue preference (e.g., favorable and unfavorable residues on interfaces). Our multi-interface properties and the generic profiles of binding interfaces can be combined to facilitate many applications such as protein function analysis [23]–[25], protein engineering [26], [27], drug development [28], [29].

To this end, we have compiled a representative data set of multi-interface domains from PDB [30]. Based on this comprehensive data set, graph theory and algorithms are applied to construct interface graph, to mine fingerprints of multiple interfaces, and to explore relations within multiple interfaces as well as associations between interfaces and their molecular functions. All the data sets and supplementary files are available at http://sunim1.sce.ntu.edu.sg/~s080011/metp/index.html.

## Materials and Methods

**Figure 3:**
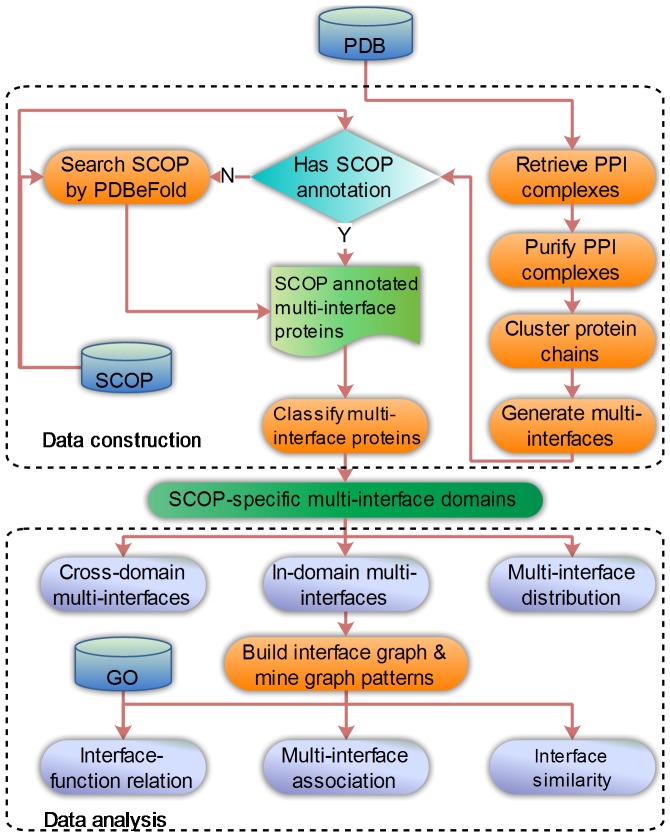
Flowchart of multi-interface protein data set construction and data analysis.

Our data and method are outlined in Figure 3, in which the upper part shows the steps for the data set construction, while the lower part is a diagram of our data analysis.

### Compilation of multi-interface proteins

We compile multi-interface proteins in the following four steps:

- Retrieve PPI (Protein-Protein Interaction) complexes from PDB;
- Preprocess PPI complexes by eliminating transformed chains and short chains;
- Cluster protein chains by sequence similarity;
- Identify representative multi-interface proteins from each protein cluster.

#### Retrieve PPI complexes

We retrieve those PDB complexes produced by X-Ray crystallography that satisfy the following criteria: (i) Their macromolecule type is protein but not DNA or RNA. We exclude DNA/RNA because the difference between protein-protein binding and protein-DNA/RNA binding may cause confusion in defining multi-interface proteins. (ii) The number of protein chains within each biological unit in one complex is bigger than 1. (iii) The chain length is larger than or equal to 30. (iv) Their X-ray resolution is better than 3.0 Å. Using these selection criteria, 35,760 PDB entries containing 109,672 protein chains are obtained.

#### Preprocess PPI complexes

A few chains in the PDB complexes have transformed coordinates (458 out of 109,672 chains in our data set). Hence the structural relation within these chains or between these chains and the other chains in the same complex may not be correctly represented by their original coordinates. We thus remove those protein chains with a non-identity transformation matrix to clean the noise in the final data set. For example, there are two chains (A and B) with transformed atom coordinates in biomolecule 2 of PDB entry 3HZN, which contains a total of four chains (A, B, C and D); thus the interaction between chain A and chain D is incorrect without coordinates transformation. Besides excluding transformed chains, those protein chains with less than 30 amino acids are also removed from each PDB complex. With the removal of transformed chains and short chains, some chains do not have interaction partner anymore. These chains or complexes are further eliminated from the data set. Finally, a total of 24,664 PDB entries with 87,395 protein chains are used for analysis.

#### Cluster similar chains

The same protein chain interacting with different protein chains can be included in different PDB entries. Therefore, different instances of each chain have to be clustered together to identify multiple interfaces. We cluster similar protein chains by the following steps: (i) For each chain in the compiled data set containing 87,395 protein chains, we search the other 87,394 chains using BLAST [31] with e-value of 0.001, and store the similar chains satisfying the e-value criterion. (ii) We build a graph 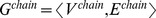 for the entire set of 87,395 chains based on their sequence similarity, and cluster the graph 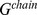 into subgroups based on connected components—*i.e.*, each connected component forms a cluster. Sequence similarity 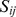 between two chains 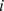 and 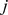 is defined as 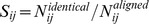, where 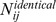 is the number of identical aligned residues between chains 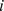 and 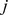, and 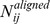 is the length of alignment between chains 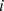 and 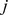. The alignments used are the ones produced by BLAST. Nodes in 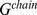 are protein chains, and edges are similar chain-pairs with mutation rate lower than or equal to 2.5%. Pair-wise sequence similarity is calculated based on aligned length instead of the whole sequence length. This strategy can detect multi-interface domains included in proteins with more than one domain. Although it may lead to clustering by short peptides, 99.4% of the clusters have the minimum pair-wise sequence similarity of 0.9, indicating that this situation rarely happens in our data. Besides, almost all the clusters have the minimum pair-wise sequence similarity of 0.9, confirming that our data is nearly free of chaining events due to using a single-linkage clustering algorithm. This yields 13,295 clusters. 11,681 clusters among these have more than one chains, indicating that each of the 11,681 clusters has at least two potential interfaces. In this study, sequence similarity instead of structure similarity is used to cluster protein chains. This is because structure similarity may introduce other proteins into one cluster due to different proteins may have similar structures. Obviously, different proteins cannot be grouped together to identify multiple interfaces.

#### Identify representative multi-interface proteins

For the 11,681 clusters, we first identify the interface residues for each chain of every cluster. Interface residues in the surface of a chain are determined by using a Euclidian distance of 5 Åwhich is commonly used to determine inter-chain contacts [32]. A residue is considered as an interface residue if at least one of its heavy atoms is within 5 Å to a heavy atom of its ligand residue. The threshold of 5 Å is commonly used for determining interface residues from protein quaternary structures. Protein chains that are near each other in 3D space but not in the same biological unit are excluded from this interface residue determination procedure. For instance, the six chains in PDB entry 1UT1 are A, C, and E of bio-molecule 1 and B, D, and F of bio-molecule 2. By definition, we only consider the interfaces between chains of bio-molecule 1 or bio-molecule 2 but not those between bio-molecule 1 and bio-molecule 2. Since the same protein chain can be included in different PDB entries, redundant interfaces may exist in one cluster. Therefore, we further calculate the similarities between different interfaces in one protein cluster to eliminate duplicates or similar interfaces. This is done in five steps: (i) Align all the chains together in one cluster using ClustalW [33]. (ii) Adjust position label of interface residues for all the interfaces in each cluster according to the multiple sequence alignment. This is necessary as the same chain can be numbered diversely in different entries. (iii) Calculate pair-wise interface similarities. Interface similarity is defined as 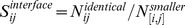, where 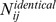 is the number of identical aligned interface residues and 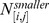 is the cardinality of the smaller of the two sets of interface residues between interface 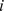 and interface 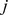. Here, two interface residues are considered as identical if they have the same amino acid type and the same position according to the multiple sequence alignment. (iv) Construct a graph for all the interfaces in a cluster, in which nodes are interfaces and edges represent similar interface pairs with similarity equal to or lager than 0.8. (v) Determine connected components for every interface graph and choose one representative interface for each component. The representative interface is chosen based on the best X-ray resolution of all the chains in that connected component. In case more than one chains have the same best resolution, then the first one encountered is chosen. Finally, all interfaces in each representative protein of one cluster will be mapped to one of these representative proteins with the best resolution. Following these steps, we have collected 5,222 multi-interface proteins for further use in our in-depth data analysis. For the other 6,459 (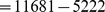) clusters, each interface graph is actually a clique—*i.e.*, all the chains in a cluster share a similar interface.

### Aggregation of multi-interface proteins

To explore which domains have multiple interfaces as well as their distributions in PDB, we aggregate all these 5,222 multi-interface proteins according to their structural annotations.

Protein structural annotations are obtained through following steps. First we directly retrieve each multi-interface protein's structural annotations from SCOP [1]. Then, for those proteins that do not have SCOP annotations, we employ PDBeFold [34] to search for annotations of similar proteins stored in SCOP. Among the results generated by PDBeFold for a given protein, we chose the one with the best Q-score as the target domain and retrieve the complete information of the protein containing this domain from SCOP.

Based on structural annotations, multi-interface proteins are further aggregated into several groups in accordance with SCOP classification, as per the following steps: (i) Align each multi-interface protein sequence to its target domain sequence. (ii) Categorize each interface to a domain by the interface residues' position and domain range. If the entire set of interface residues fall into one domain for a given interface then it is annotated by that domain identifier; otherwise, multiple domain identifiers are tagged to that interface. (iii) Aggregate multi-interface proteins into clusters at different SCOP classification levels, *i.e.*, class, fold, superfamily, family, and domain, according to their annotations.

### Construction of interface graphs

Interface analysis is carried out on their fingerprints, where each fingerprint is represented by closed frequent interface residue contacting graph. Each interface in the 5,222 multi-interface proteins is represented as a graph 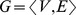, where 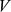 is a set of interface residues and 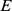 is a set of edges representing the spatial closeness between residue pairs. Edges in each interface satisfy two criteria: the Delaunay triangulation rule and the distance threshold of 5 Å. Delaunay triangulation, aiming to maximize the minimum angle of all triangles in the interface graph, is perceptually more meaningful and widely used to build biological structural networks [35], [36]. While distance threshold is used to eliminate those contacts constructed by Delaunay tessellation but are improbable real contacts in practice. Each interface graph is built according to the following three steps: (i) Retrieve all heavy atoms' 3D coordinates for each surface residue stored in its PDB file and transform them into Qhull [37] input format. Only heavy atoms are considered because very few hydrogen atoms' coordinates are reported. (ii) Construct atom contacting graph by Qhull [37]. (iii) Upgrade atom contacting graph into residue contacting graph and pick out interface contacting graph from residue contacting graph. Upgrading is conducted as follows: atom contacts in the same residue are ignored and atom contacts between different residues are kept. For multiple contacts between two residues produced by upgrading, they are further merged together into one contact. That is, connection between two residues is captured in the resulting interface residue contacting graph but not the number of connections between them.

### Mining structural patterns from in-domain multiple interfaces

Since every interface is represented by a graph, all interfaces in a domain form a graph database. We are interested in closed frequent subgraphs, paired cooperative subgraph sets and distinctive subgraph sets between interfaces to uncover fingerprints for interfaces and to identify relations between multiple interfaces of one domain.

We introduce some additional notations. A graph 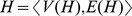 is a subgraph of a graph 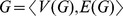, denoted by 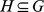, if 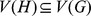 and 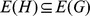. The support of a subgraph 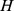 in a graph database 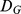 is the number of graphs in 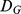 that contain 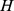 as a subgraph. A subgraph 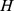 is said to be frequent in 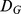 under a given support threshold count 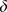 if the support of 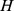 is at least 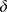. A frequent subgraph 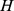 is closed in 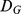 if 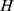 cannot be extended by any additional node or edge without changing its support in 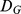. A paired cooperative subgraph set 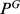 is a paired set of graphs 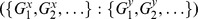 such that 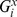 occurs in interface 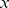 and 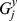 occurs in interface 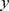 of the same domain simultaneously. A distinctive graph set 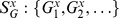 says that each graph 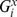 in 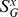 only occurs in the interface 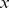 of a given domain but never occur in other interfaces of the same domain. The detailed procedures for mining these graph patterns are described below.

Closed frequent subgraphs of one interface cluster, which are deemed as fingerprints of one interface, are mined from an interface graph data base, where the graph database is a set of interface residue contacting graphs in one domain. Closed frequent subgraphs instead of frequent subgraphs are mined since we aim to figure out the largest structures that can be used to identify a specific interface for a given domain. To explore fingerprints of each interface, we first align all protein's structures together by the CE algorithm [38] for each domain and then cluster the interfaces together based on their spatial similarity. Subsequently, fingerprints for each interface are mined by ParMol [39] with the FFSM algorithm [40]. The local support of mining fingerprints is set to 20% in this study, thus the global support 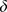 is set as the number of graphs times 0.20.

Interface relation in one domain is described by paired cooperative graph sets and distinctive graph sets. Paired cooperative graph sets between two different interfaces in a domain are identified using the following steps: (i) Build graph database for each set of interfaces. (ii) Mine closed frequent subgraphs by ParMol [39] for each graph database with local support of 20%. (iii) Transform sets of closed frequent subgraphs into a transactional data set and mine closed frequent item set with LCM [41]. In this transactional data set, each transaction is a closed frequent subgraph and the items in each transaction are the different interfaces. With these steps we can identify all paired co-existing subgraph sets in a sets of interfaces. Exploring distinctive relations between interfaces is much easier than mining cooperative relations. To obtain a unique set of fingerprints for an interface of a multi-interface domain, we first simply mine all closed frequent subgraphs from the set of interfaces, and then pick out those graphs that belong to this interface but not to others. These selected fingerprints then form the unique fingerprints of this interface. Cooperative relation tells the connection between two different interfaces and distinctive relation discriminates one interface type (function) from other interfaces. Distinctive relation can be used to identify specific interface and cooperative relation can be useful to infer new interfaces based on known interfaces.

## Results

In this section, we present results to show the distributions of multi-interface proteins and multi-interface domains, fingerprints of interfaces, cooperative and distinctive relations between multiple interfaces, associations between interfaces and molecular functions, and some properties of cross-domain interfaces.

### Distributions of multi-interface proteins and multi-interface domains

The 87,395 protein chains are grouped into 13,295 clusters according to their sequence similarities generated by BLAST [31]. For the 13,295 clusters, there are 5,222 multi-interface proteins with a total of 15,345 interfaces—*i.e.*, 3 interfaces each on average. We note that the number of multi-interface proteins differs with regard to the change of interface similarity threshold due to promiscuity of interface [42]. For example, Kim and colleagues identified 873 multi-interface proteins based on structural exclusion in which similarity threshold is 0 [2], and Kar and coworkers only obtained 79 cancer-related multi-interface proteins with threshold of 0.2 [43]. The distribution of multi-interface proteins of our data in terms of the number of interfaces of a protein is shown in Table 1, indicating that most of the multi-interface proteins have a very small number of interfaces and only a few of them have more than 5.

**Table 1:** Multi-interface protein distribution in terms of the number of multiple interfaces.

# interfaces	# protein chains
2	2735
3	1349
4	608
5	241
6	119
7	62
8	45
9	28
10	11
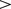 10	24

**Figure 4:**
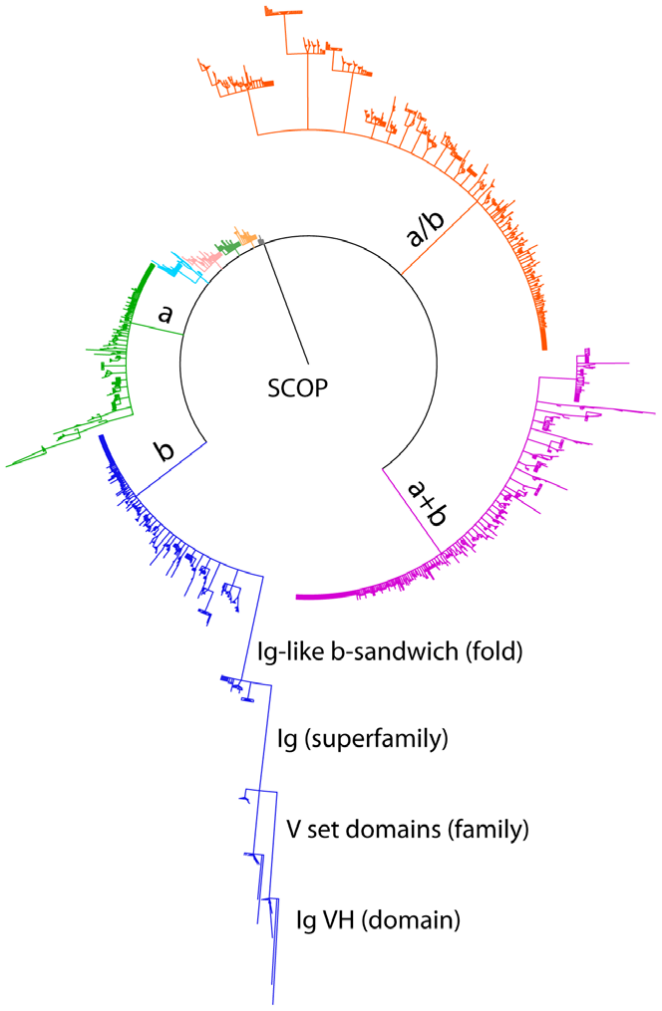
Multi-interface domain distribution at different SCOP levels. Length of lines represents the normalized number of multi-interface proteins at each classification level, and multiple lines under the same level of one cluster represent different sub-clusters. The clusters are organized as a rooted tree structure from a higher level to a lower level, and the clusters at the same level are plotted in the clockwise descendant order. Here *a* represents 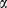 and *b* represents 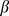.

Figure 4 is produced by PHYLIP [44] based on 2,517 of the 5,222 multi-interface proteins that have SCOP annotations. It shows the number distribution of multi-interface proteins at different SCOP classification levels. Obviously, multi-interface domains can appear in a broad range of clusters in terms of SCOP classification. Among all the eleven classes in SCOP, 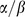 proteins, 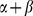 proteins, all-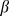 proteins, and all-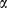 proteins account for 90.3% of all the multi-interface proteins. Figure 4 also indicates that all-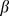 proteins, or at least part of them, are less conservable since they have the largest number of multi-interface proteins in one domain. It can be also seen that multi-interface proteins with a large variability tend to aggregate to a small number of clusters instead of uniformly spread out to each cluster as shown in Figure 4.

Table 2 gives the distribution of the 2,517 multi-interface proteins at different levels of SCOP classification. The complete number of sub-levels for each classification level is retrieved from SCOP [1], while the number of sub-levels with multiple interfaces for each level is determined by the number of multi-interface domains “upgraded” from the domain level to the class level. It can be seen from Figure 4 that, while multi-interface proteins exist over all classes of SCOP classification, they clearly favor a few of the sub-levels. In particular, although there are more than 110,000 domains with annotation in SCOP [1], only a very small proportion of these domains (1,730/97,178) have the multi-interface property. This phenomenon also suggests that all biological processes have their own small set of pivotal proteins [45].

**Table 2:** Distribution of multi-interface domains at different SCOP classification levels.

class	fold		superfamily		family		domain	
	N 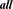 •	N 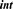 °	N 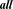	N 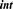	N 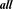	N 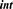	N 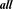	N 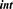
All 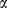 proteins (349)†	259	89	459	128	772	174	-‡	255
All 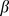 proteins (577)	165	85	331	138	679	207	-	336
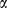 and 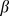 proteins ( 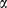 / 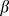 ) (684)	141	91	232	137	736	293	-	527
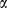 and 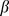 proteins ( 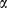 + 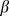 ) (662)	334	161	488	209	897	318	-	476
Multi-domain proteins (45)	53	21	53	21	74	26	-	36
Membrane and cell surface proteins (53)	50	24	92	35	104	38	-	43
Small proteins (67)	85	24	122	29	202	38	-	57
Total	1087	495	1777	697	3464	1094	97178*	1730

• number of clusters under the given SCOP classification level;

° number of clusters that have multi-interface proteins under the given SCOP classification level;

† number of protein chains that have multi-interfaces;

‡ data is not available in SCOP;

* the total number of domains is 97,178 in SCOP version 1.73, but the total number of domains listed above is slightly smaller since there are still four classes with very few number of domains are not shown here.

SCOP version 1.73 instead of 1.75 is used in this study because the PDBeFold [34] is based on SCOP version 1.73, which is used to search SCOP to get similar domains for a given protein.

Interfaces between proteins can be categorized into homo-oligomer, homo-complex, hetero-oligomer and hetero-complex in terms of sequence similarity and interaction lifetime [46]. Therefore, we classified the interactions of the 5,222 multi-interface proteins into the aforementioned four types by the method described in [47]. Then, we analyzed the preference for each interaction type, with the preference defined as 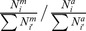, where 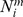 is the number of multi-interface protein interactions of type 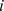 and 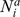 is the whole number of interactions of type 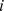 in our data. This was followed by mining fingerprints of every interaction type and exploring relations between fingerprints of different interaction types. According to preferences shown in Table 3, proteins with multiple interfaces are favored in homo-interactions (homo-oligomer and homo-complex). Fingerprints mined from each type of interaction with a minimum frequency of 5% show that homo-oligomers have 96 non-trivial fingerprints and that the same number is significantly lower for hetero-complexes (9). This indicates that interfaces of homo-oligomers share some common structural patterns, although homo-oligomers are different from each other, and interfaces of hetero-complexes rarely have recurring structural patterns. Surprisingly, despite only 9 fingerprints mined from hetero-complexes, 4 of them are isomorphic to (other 4) fingerprints (of the 96 mined) from homo-oligomers. The *p*-value of this number of isomorphic fingerprints against randomly generated graph pairs is 6.7e-4. This shows that sharing of interface patterns between homo-oligomers and hetero-complexes is significantly more frequent than expected by chance. Due to a lack of homo-complex and hetero-oligomer interactions in multi-interface proteins, we cannot get reasonable results from these two types of interactions.

**Table 3:** Preference of multi-interface protein interactions of four interface types.

Interface type	# interaction	# multi-interface interaction	preference
Homo-oligomer	647	127	1.42
Homo-complex	13	3	1.67
Hetero-oligomer	38	4	0.76
Hetero-complex	6695	890	0.96

### Analysis on multiple interfaces within the same domain

The top ten multi-interface domains with the largest numbers of proteins are shown in Table 4. Unexpectedly, domains from the immune system have the most number of multi-interface proteins. This observation in part can be explained by the fact that a small portion of hypervariable regions exist in these proteins [48]. That is, although these proteins have the same domain, they do contain different interfaces due to mutations occurring in the hypervariable regions.

**Table 4:** Top ten multi-interface domains with the largest numbers of proteins.

Domain name	# multi-interface proteins	avg # interfaces
Ig heavy chain variable domain, VH	43	2.4( 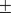 0.7)
Ig light chain 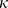 variable domain, VL- 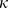	35	2.5( 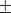 0.8)
Ig heavy chain 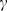 constant domain 1, CH1- 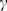	28	2.3( 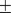 0.6)
Ig light chain 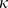 constant domain, CL- 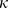	18	2.4( 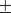 0.6)
Hemoglobin, beta-chain	17	2.8( 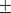 0.9)
Proteasome beta subunit (catalytic)	17	**7.6(** 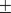 **3.2)**
T-cell antigen receptor	16	3.1( 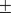 1.6)
Hemoglobin, alpha-chain	14	3.1( 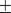 0.5)
Nucleoside diphosphate kinase, NDK	13	2.9( 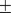 0.3)
Dodecameric ferritin homolog	12	5.4( 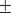 1.4)

Table 4 also unveils the wide coverage of biological functions played by some domain. For example, the proteasome beta subunit (catalytic) domain has around seven different interfaces which are involved in different biological processes. This is quite different from the Ig VH domain with the typical two interfaces playing the Ig VL protein binding role and the role of antigen recognition.

Since there are as many as 1,730 multi-interface domains—see Figure 4 and Table 2—and exploring properties of every multi-interface domain is not our purpose in this study, we undertake analysis on two domains: Ig VH domain and proteasome beta subunit domain. The former is contained in the largest number of multi-interface proteins and the latter has many binding sites besides a sufficient number of multi-interface proteins holding this domain.

#### Fingerprints of interface

Given a set of multiple interfaces in a domain, we fish out interface-specific fingerprints by the mining of closed frequent subgraphs (substructures) from the corresponding interface graph database. These frequent substructures capture the natural organizations of interface residues. In the past, frequent sub-structures have been successfully applied to study protein structure and function [49].

**Figure 5:**
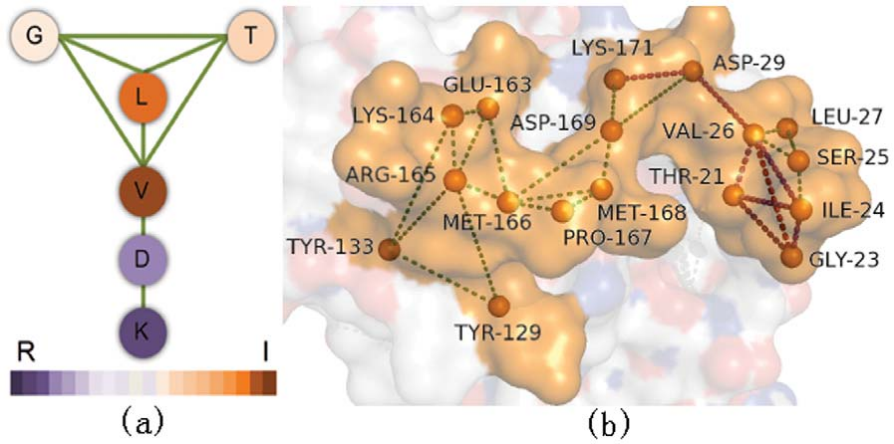
Amino acid distribution of the antigen-binding interface and the protein-binding interface in the Ig VH domain.

**Figure 6:**
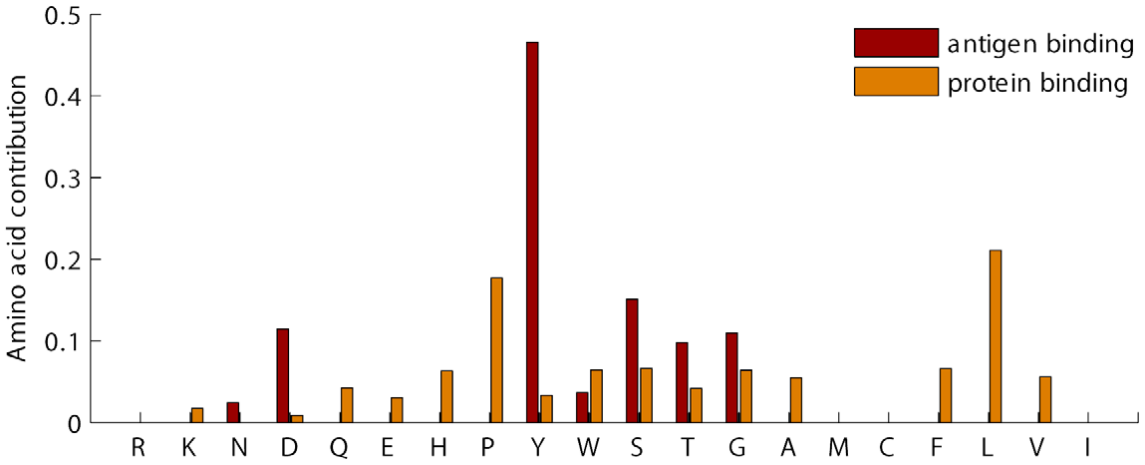
Amino acid distribution of the six binding sites in the proteasome beta subunit domain. *bs* means binding site.

Our experiments on identifying interface fingerprints are carried out on Ig VH domain and proteasome beta subunit domain separately. Generally, we obtained a great number of non-trivial closed frequent substructures for each interface of the two domains. Full results are provided in Supplement Data S1. We then examine whether these graph patterns (fingerprints) are domain-specific or not. To this end, we compared fingerprints between interfaces in various dimensions. First, we directly compared residue composition of different interfaces. The detailed results are shown in Figure 5 and Figure 6 for Ig VH domain and proteasome beta subunit domain, respectively. It is obvious that residue preferences are divergent for different interfaces. Second, we explored fingerprint isomorphism between different interfaces. Not surprisingly, we identified just one isomorphic fingerprint between the interfaces of Ig VH domain and very few isomorphic fingerprints between interfaces of proteasome beta subunit domain shown in Table 5. Based on these observations, we can safely draw the conclusion that the fingerprints are interface-type specific and they indeed can distinguish different interfaces of one domain.

**Table 5:** Number of isomorphic fingerprints between different interfaces within the proteasome beta subunit domain.

	Binding site A	Binding site B	Binding site C	Binding site D	Binding site E	Binding site F
	(271†)	(49)	(685)	(21)	(251)	(237)
Binding site A		7 ( 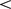 5.0e-4*)	10 (2.0e-3)	0 (7.3e-1)	6 (1.9e-2)	5 (1.3e-2)
Binding site B	7 ( 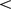 5.0e-4)		3 (7.6e-2)	0 (9.1e-1)	0 (5.0e-1)	4 (1.0e-3)
Binding site C	10 (3.3e-3)	3 (8.7e-2)		2 (9.1e-2)	17 ( 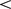 5.0e-4)	9 (5.0e-4)
Binding site D	0 (7.4e-1)	0 (9.1e-1)	2 (8.3e-2)		1 (2.4e-1)	0 (8.3e-1)
Binding site E	6 (1.3e-2)	0 (5.2e-1)	9 (2.0e-3)	1 (2.2e-2)		3 (1.0e-1)
Binding site F	3 (3.3e-2)	4 ( 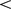 5.0e-4)	4 (5.3e-2)	0 (8.3e-1)	3 (9.0e-2)	

Values in the upper triangle are calculated based on interface overlapping threshold of 0.8, while the lower triangle values are computed with no overlapping residues between interfaces.

† Number of fingerprints, and

* *p*-value. *p*-value is calculated against randomly generated graphs based on fingerprints of two arbitrary binding sites 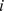 and 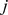 with equal graph size and number of edges.

Node labels of generated graphs are determined based on amino acid frequency.

**Figure 7:**
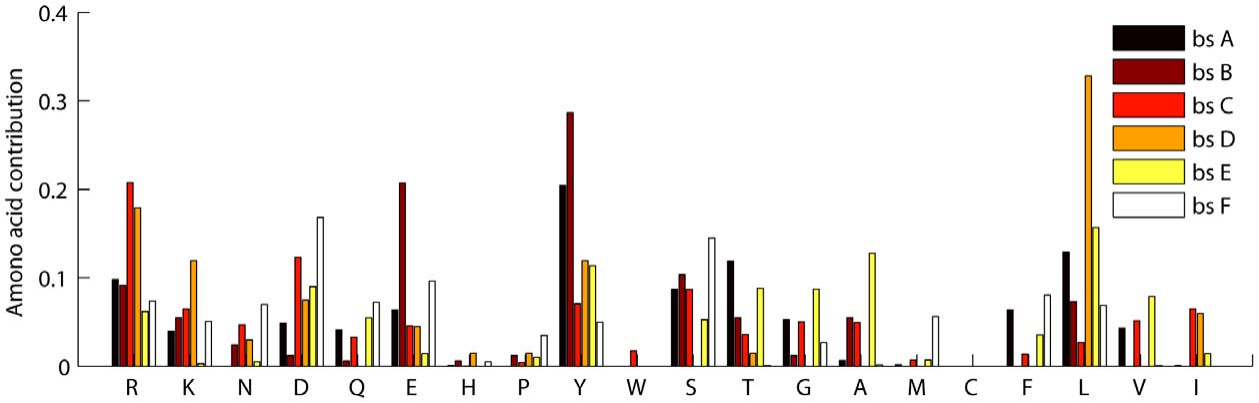
An example of a fingerprint in the proteasome beta subunit domain. (a) is a diagram of the given fingerprint, where the filled circles represent the interface residues, and the lines represent the contacts. Color and color shade represent residue hydrophobicity index defined by Kyte and Doolittle [54]. (b) is the real structure of (a) presented in PDB entry 3NZX. The dashed lines represent the contacts determined by Delaunay triangulation, and the highlighted lines are the contacts shown in (a).

Figure 7 shows an example of a fingerprint in proteasome beta subunit domain. The real data of this structure contained in PDB entry 3NZX is shown in Figure 7(b). With hydrophobicity information labeled to each node in this structure, we found that the center of binding sites is filled with hydrophobic residues which is surrounded by hydrophilic residues as can be observed from Figure 7(b). This result is consistent with the previous wet-rim-dry-core observation of binding site [50].

#### Cooperative and distinctive relations between multi-interfaces

Closed frequent substructures characterize the common organization of interfaces in a domain. However, they cannot reveal the relations between multiple interfaces in that domain. In addition, one domain is a perfectly assembled structure constituted by all kinds of residues. It is believed that protein structures are not randomly assembled together and they should incorporate some cooperative or competitive relations [51], [52]. Thus we wonder whether correlations exist between different interfaces in a domain. To this end, co-existing paired fingerprints in different interfaces are used to describe cooperative relations, and unique fingerprints are employed to depict distinctive relations between multiple interfaces in one domain. Please refer to the methods section for detailed descriptions of these ideas.

**Figure 8:**
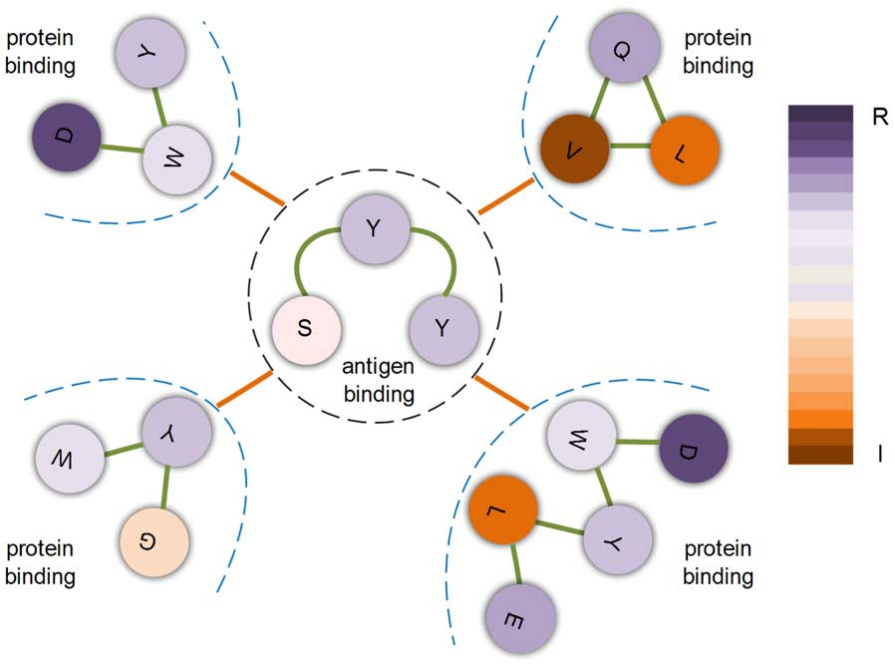
An example of a pair of fingerprints from multiple interfaces within the Ig VH domain. The dash line separated structures are fingerprints, and the solid orange lines between these fingerprints represent cooperative relations between them. The filled circles represent interface residues and the solid green lines represent residue contacts. Color of residues indicates their hydrophobicity.

Co-existing paired fingerprints somehow could reveal reenforcement of interface residues from a physicochemical perspective which could help wet-lab experiments. Our experimental results reveal that a large number of co-existing fingerprints of different interfaces exist both in Ig VH domain and proeasome beta subunit domain. The complete co-existing paired fingerprints of this two domains are presented in supplement Data S2. An example of co-existing paired fingerprints in Ig VH domain is shown in Figure 8, which is contained in two thirds of all multi-interface proteins of this domain.

Unique fingerprints of each interface are the signatures that distinguish one interface from the other interfaces in the same domain. Observations show that, besides cooperative fingerprints, a large number of fingerprints are interface-specific as shown in Table 5. Statistical analysis shown in Table 5 also reveal that, although very few isomorphic fingerprints can be identified from different binding sites of proteasome beta subunit domain, some pairs of binding sites indeed have significant number of common fingerprints as indicated by the very confident 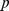-values, which do consolidate the argument that cooperative relation exists between different binding sites of proteasome beta subunit domain.

Overlapping residues between interfaces distort the study of co-existing paired fingerprints and common fingerprints between interfaces. Thus we further investigated the influence of overlapping, measured by Jaccard index, between multiple interfaces. Our experimental result on Ig VH domain shows that overlapping between antigen binding interface and protein binding interface is 0.04
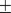
0.05 which means the overlapping has marginal effect on cooperative fingerprints analysis. Besides, cooperative fingerprints in each pair are quite different, indicating they are not from the overlapping region of interfaces, as shown in Figure 8. For control, we carried out isomorphic fingerprints testing on non-overlapping interfaces and interfaces with overlapping threshold of 0.8 on proteasome beta subunit domain. Results are shown in Table 5. The change of *p*-values before and after the removal of overlapping residues implies a little influence of the overlapping residues, but the very slight change indicates that cooperative fingerprints are not caused by overlapping, as shown in Table 5.

Co-existing paired fingerprints and distinctive fingerprints shown in our data do reveal that interface is distinguishable and cooperates with each other to some extent in the same domain.

#### Association between interface's fingerprints and its function

The organization of interface residues determines its capable binding partners, which further specifies its associated molecular function. Here, we conduct case studies to examine the existence of association between multiple interfaces and their biological functions. We take the following two domains as examples: Ig VH domain and the proteasome beta subunit domain.

Ig VH domain has, as annotated by GO [5], two molecular functions: protein binding and antigen binding, while the functions of proteasome beta subunit domain include: threonine-type endopeptidase activity, peptidase activity, hydrolase activity, endopeptidase activity, protein binding, endopeptidase activator activity, RNA binding, and NF-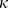
B binding. Since the functions are assigned by GO at the domain levle instead of at the level of interface, we further utilize structural information and alignment as well as PDB remarks and description to manually tag the functions to their corresponding interfaces. For instance, the functions for the interfaces of Ig VH domain are determined by their locations. The criteria is that an interface from Ig VH domain is considered to play the function of antigen binding if it situated in the complementarity determining regions of this antibody, otherwise it is considered as having the function of protein binding. By analyzing the multi-interface profiles in the above section, we found that the interfaces of these two domains are distinguishable by their fingerprints. Although a few of the fingerprints are shared by multiple interfaces, lots of them are unique to their interface type. For example, 73 and 1182 fingerprints are identified for the antigen binding interface and protein binding interface from Ig VH domain, respectively. But there is only one isomorphic fingerprints between the two sets of fingerprints. Hence almost all of the fingerprints belonging to the antigen binding interface and protein binding interface can be used to specify their functions, except the common one. Regarding the proteasome beta subunit domain, the number of fingerprints for the six interfaces ranges from 21 to 685 as shown in Table 5; but the largest number of isomorphic fingerprints between each pair of these interfaces is only 17. Therefore, it is capable of using the unique fingerprints of each interface in this domain to specify its biological function. Data that display function-type specific interfaces for these two domains are shown in supplement Data S3.

Based on the above observations, we can see that large numbers of unique fingerprints exist in the specific interfaces of Ig VH domain and those of the proteasome beta subunit domain. It suggests that the unique fingerprints of the interfaces in these two domains can be used to determine their biological functions. This observation is interesting, however, it is concluded just based on the two case studies. It does not cover some other situations, including different domains with the same function and the same domain with more than one functions. Thoroughly exploring the association between interfaces and their functions under various situations is difficult and time costly. One important reason is that the correlation between the interfaces and functions is not a bijection. For example, an antigen can have multiple epitopes binding to different antibodies although the same function is annotated. Therefore, more efforts and data are needed to annotate a function to an interface. Another important reason is as follows. For each domain of a protein, those proteins containing the same domain are obtained by searching the whole PDB, and then the interfaces of this domain are determined based on the structural information. This is followed by identifying different interfaces and retrieving function annotations for this domain. Subsequently, functions are mapped to interfaces by using physicochemical information. Since the number of domains is huge (more than 110,800 domains are available in SCOP, which only accounts for about half of the PDB entries) and multiple binding partners are possible, the process of mapping functions to interfaces is very time costly and laborious. Because of these limitations and difficulties, statistically reliable association between interfaces and specific functions of a multi-interface domain will be one of our future works to extend the current study.

#### Interface fingerprints between different domains

**Figure 9:**
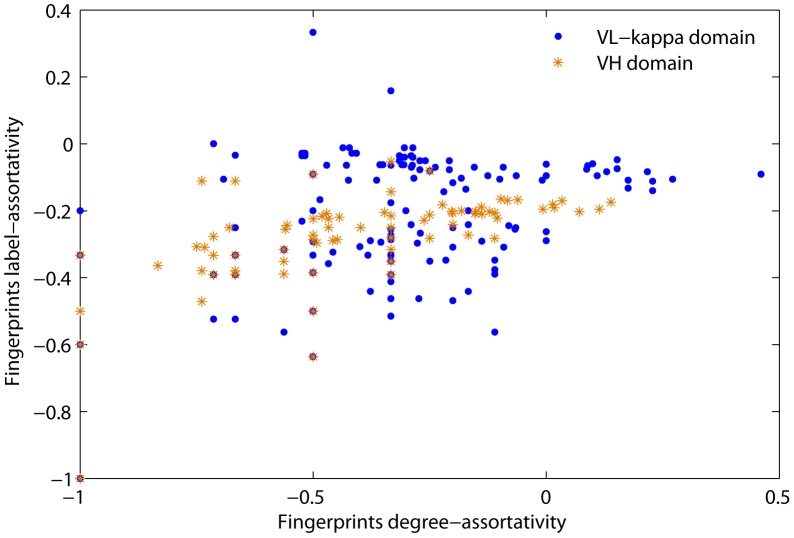
Fingerprints assortativity of the Ig VH domain and Ig VL-kappa domain.

We have identified interface fingerprints and analyzed their similarities between interfaces within a domain. But it is still unknown whether these fingerprints are domain-specific or not for multi-interface domains. To address this issue, we take a small trick of comparing the similarity of fingerprints of two very similar domains in the same protein family. The assumption is that if the similarity is low for the two very close domains, then the similarity should be much lower for two randomly selected domains. Based on this assumption, we analyzed the fingerprints similarity between the Ig VH domain and Ig VL kappa domain which have similar number of multi-interface proteins, and both belong to the V-set domain family. We employ graph assortativity to measure the similarity between different sets of fingerprints. Assortativity is a preferred metric to quantify the equivalence of a network's nodes and connections in graph theories. Assortativity between fingerprints of this two domains is calculated using NetworkX [53]. Figure 9 shows the assortativity of fingerprints in the Ig VL-kappa domain and Ig VH domain. The null hypothesis says that the assortativity of fingerprints between the two domains are the same. Then we calculated the t-test *p*-value of graph label-assortativity (residue type) and graph degree-assortativity of the fingerprints from the Ig VL-kappa domain and Ig VH domain. The 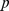-values 1.7e-4 and 1.0e-2 under the two tests suggest the significant difference of the fingerprints between Ig VL-kappa domain and Ig VH domain.

However, difference between the fingerprints of two domains could be induced by the fingerprint topology or the number of fingerprints with their combinations. Therefore, we conducted additional experiments to understand the influence of fingerprint topology and fingerprint volume on analyzing fingerprints' domain-specificity by exploring the difference within randomly generated fingerprints and by examining disparity between subsets of fingerprints of the two domains. To explore the effect of fingerprint topology on this domain-specificity, we randomly generated two sets of fingerprints with one having an equal number of fingerprints to that of Ig VL-kappa domain and the other having the same number as that of Ig VH domain. The 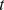-test 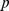-values of degree-assortativity and label-assortativity between the two sets of randomly generated fingerprints are 8.2e-1 and 9.2e-1, respectively. These insignificant 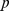-values indicate that the randomly generated fingerprints are very similar; and thus this test verifies that the difference between the fingerprints of Ig VL-kappa domain and Ig VH domain is not trivial. To examine the influence of fingerprint volume on this domain-specificity, we sampled a small set of fingerprints (50, in our experiment) for each domain, and calculated the disparity between them in terms of isomorphic fingerprints. This sampling with replacement was carried out for thousands of times and were subsequently used to compute the 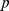-value of the difference between subsets of fingerprints of the two domains. The significant 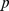-value of 7.0e-2 suggests that the difference between the two domains is not dominantly caused by the large number of fingerprints and their combinations. Based on the above analysis, we can conclude that the fingerprints of multi-interface domains are domain-specific. As this study focuses on multi-interface domains, the domain-specificity is not examined for the single-interface domains.

### Profiling of domains with cross-domain multi-interface

**Figure 10:**
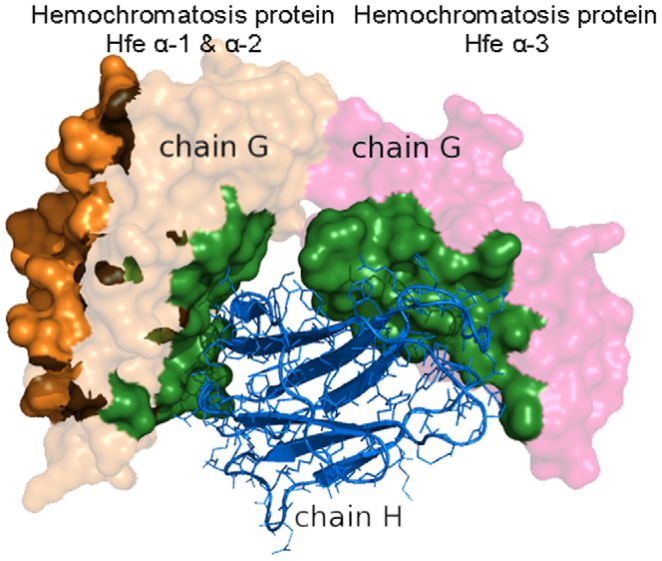
An example of a cross-domain interface in a multi-interface domain. The two interfaces are colored by orange and forest, respectively. The forest colored interface is a cross-domain interface formed by the interaction between chain H (rendered by surface) and chain G (rendered by cartoon). Chain H has two domains, which are Hemochromatosis protein Hfe 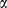-1 and 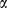
2 domain (the left part of chain H) and Hemochromatosis protein Hfe 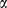-3 domain (the right part of chain H).

**Figure 11:**
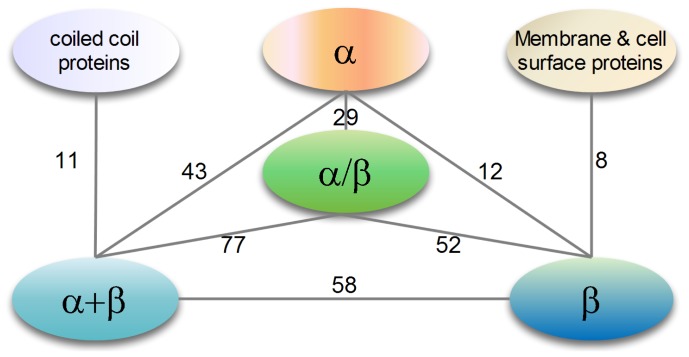
Distribution of cross-domain interfaces at various SCOP class levels. A line between two classes means a cross-domain interface in these two classes. The weight on each line indicates the number of instances that have cross-domain interfaces in our data set. Cross-domain multi-interfaces with very small numbers are not shown here.

In protein-protein interacting complexes, most of their interfaces are located within one single domain. But there are still a few interfaces that spread over other domains; these are named cross-domain interfaces in this study. Our results show that among the 2,517 multi-interface proteins with SCOP annotations, 301 proteins contain cross-domain interfaces. The detailed result is shown in supplement Data S4. This seems a bit surprising as it is usually believed that domain is the basic functional unit in cell. This, however, is not always true as in some circumstances they have to be combined together to play a certain function. Figure 10 shows an example of a cross-domain interface in PDB entry 1DE4, in which the protein-binding interface in chain G spans over Hemochromatosis protein Hfe 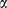-1, 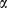-2, and 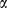-3 domains. The numbers of cross-domain interfaces in various SCOP class levels are shown in Figure 11. It can be seen that cross-domain interfaces are favored in 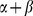 and 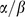 proteins. However, cooperative fingerprints between cross-domain interfaces at SCOP class level can be rarely obtained. This could be attributed to the large number of domains in each class (see Table 2) with a very small number of cross-domain interfaces (301 in total). Cross-domain interfaces between different classes are observed (see Figure 11), but the ones between the same classes do not appear in our data. Based on this observation, we can conclude that domain is mainly the functional unit, but with some exceptions.

## Summary and Discussion

Multi-interface domains have been demonstrated to have multiple non-exchangeable interfaces in every domain. In particular, this work has conducted comprehensive analysis on the following aspects of multi-interface domains: (i) which domains have multiple interfaces; (ii) what the fingerprints of the multiple interfaces are; (iii) the relations of the multi-interfaces in a domain; (iv) the associations between multi-interface and their molecular functions, and (v) profiles of cross-domain multi-interfaces. Our data is a set of 5,222 multi-interface proteins obtained from 35,760 PDB entries. Interface geometric information, graph theories, closed frequent item set mining, and association mining techniques are utilized together to reveal interface signatures, associations between multiple interfaces in a domain, and relation between interface and its molecular function. Based on our systematic analysis, we found that around 40 percent of proteins have multiple interfaces which are distributed to a very small set of domains over all available domains, and that the multiple interfaces in one domain can have the same or different function types. We observed that the multiple interfaces of these domains were distinguishable in terms of their fingerprints, which further indicated the function-specific property of these interfaces in a domain. Moreover, we observed both unique and co-existing structural patterns existing between multiple interfaces of one domain, highlighting the distinctive and cooperative relations between multiple interfaces. The number of multi-interface domain is still very large although it accounts for a very small portion in the entire number of domains. Therefore, analysis is undertaken on other selected domains. Future works include building interface-function association database to facilitate a lower level analysis and to relate specific multi-interface domains to real-life applications, for example, multiple interface predictions in antigen-antibody interactions.

## Supporting Information

Data S1 Fingerprints of interfaces of Ig VH domain and proteasome beta subunit domain.
(ZIP)

Data S2 Co-existing paired fingerprints between interfaces of Ig VH domain, and cooperative fingerprints between interfaces of proteasome beta subunit domain.
(TXT)

Data S3 Function-type specific interfaces for Ig VH domain and proteasome beta subunit domain.
(ZIP)

Data S4 Cross-domain multi-interfaces in our data.
(TXT)
